# Babam2 Regulates Cell Cycle Progression and Pluripotency in Mouse Embryonic Stem Cells as Revealed by Induced DNA Damage

**DOI:** 10.3390/biomedicines8100397

**Published:** 2020-10-10

**Authors:** Cheuk Yiu Tenny Chung, Paulisally Hau Yi Lo, Kenneth Ka Ho Lee

**Affiliations:** 1 MOE Key Laboratory for Regenerative Medicine, School of Biomedical Sciences, Chinese University of Hong Kong, Shatin, Hong Kong; tennychung@hotmail.com (C.Y.T.C.); pauly@cuhk.edu.hk (P.H.Y.L.); 2 Chinese University of Hong Kong-University of Southampton Joint Laboratory for Stem Cell and Regenerative Medicine, School of Biomedical Sciences, Chinese University of Hong Kong, Shatin, Hong Kong

**Keywords:** *Babam2*, embryonic stem cells, cell cycle, DNA damage, pluripotency, senescence

## Abstract

BRISC and BRCA1-A complex member 2 (*Babam2*) plays an essential role in promoting cell cycle progression and preventing cellular senescence. *Babam2*-deficient fibroblasts show proliferation defect and premature senescence compared with their wild-type (WT) counterpart. Pluripotent mouse embryonic stem cells (mESCs) are known to have unlimited cell proliferation and self-renewal capability without entering cellular senescence. Therefore, studying the role of *Babam2* in ESCs would enable us to understand the mechanism of *Babam2* in cellular aging, cell cycle regulation, and pluripotency in ESCs. For this study, we generated *Babam2* knockout (*Babam2*^−/−^) mESCs to investigate the function of *Babam2* in mESCs. We demonstrated that the loss of *Babam2* in mESCs leads to abnormal G1 phase retention in response to DNA damage induced by gamma irradiation or doxorubicin treatments. Key cell cycle regulators, CDC25A and CDK2, were found to be degraded in *Babam2*^−/−^ mESCs following gamma irradiation. In addition, *Babam2*^−/−^ mESCs expressed p53 strongly and significantly longer than in control mESCs, where p53 inhibited Nanog expression and G1/S cell cycle progression. The combined effects significantly reduced developmental pluripotency in *Babam2*^−/−^ mESCs. In summary, *Babam2* maintains cell cycle regulation and pluripotency in mESCs in response to induced DNA damage.

## 1. Introduction

The BRISC and BRCA1-A complex member 2 (*Babam2*) gene, also known as *BRE*, encodes a highly conserved protein in mammals. It has no identifiable functional domains but possesses two ubiquitin-conjugating enzyme family-like (UEV-like) regions [1,2]. *Babam2* is expressed as early as at the two-cell stage in pre-implantation embryos, indicating that it has functions in ESC and early embryonic developments [3]. Previously, we demonstrated the importance of BRE/BABAM2 in promoting cell cycle progression and preventing cellular senescence in somatic cells [4]. *Babam2*-deficient fibroblasts show accelerated replicative and DNA damage-induced premature senescence compared with their wild-type (WT) counterpart. A recent study also revealed the molecular role of BABAM2 in deubiquitinating and stabilizing CDC25A upon DNA damage, by physically interacting with CDC25A and recruiting ubiquitin-specific-processing protease 7 (USP7) to form a BABAM2-CDC25A-USP7 complex [5]. Notably, CDC25A is involved in cell cycle progression and BABAM2 is known to regulate p53 stability by promoting Mdm2-mediated p53 ubiquitination [6]. Moreover, several cancer cell types strongly expressed BABAM2 and overexpression of BABAM2 promoted the growth of tumors [7,8,9]. Our current understanding of BABAM2’s role in pluripotency is very limited, but OCT4 and BABAM2 are known to be co-expressed in multipotent stem cells [3]. We previously reported that knockdown of BABAM2 in multipotent progenitor cells promoted chondrogenic and osteogenic differentiation [3]. On the contrary, another study reported that silencing *Babam2* impaired mesenchymal stem cell differentiation into osteoblasts [6]. Therefore, the functional role of *Babam2* in the context of stem cells is still unclear.

The inner cell mass cells of mouse blastocysts are normally used to generate pluripotent mESCs. These cells are capable of undergoing unlimited cell proliferation without entering cellular senescence and could be induced to differentiate into all types of cells found in the mouse embryo, except the placenta. mESCs possess a unique cell cycle profile that includes a shortened G2 phase and an extended S phase, as compared with somatic cells [10,11]. In addition, they have an inactivated G1 checkpoint [12,13] that allows the rapid transition from G1 phase to S phase. This maintains mESCs’ pluripotency, and cells with DNA damaged can be rapidly removed from the cell colony via apoptosis [14,15]. It has been reported that a correctly regulated cell cycle progression is essential for mESCs to maintain their pluripotency and genomic stability, especially following DNA damage [13]. Cell cycle dysregulation in stem cells can contribute to developmental defects and oncogenesis, so it must be precisely controlled in mESCs at the molecular level. CDK2 acts as a critical effector of G1/S cell cycle progression, and high CDK2 expression in mESCs is essential for the maintenance of the short G1 phase [10,11,14]. Inversely, low CDK2 expression increases the number of cells at G1 phase and a decrease at S phase [14]. Notably, inhibition of *Cdk2* leads to a loss of pluripotency-associated gene expression including *Oct4*, *Sox2*, and *Nanog* but an increase in expression of differentiation-associated genes [14,16]. Moreover, CDK2 readily phosphorylates and stabilizes OCT4, SOX2, and NANOG proteins [16,17]. Therefore, CDK2 is essential for both promoting rapid G1/S cell cycle progression and maintaining the developmental pluripotency of mESCs.

p53 is capable of binding to the *Nanog* promoter to repress *Nanog* transcription [18]. This is the reason why p53 expression is maintained at minimal levels in order to maintain pluripotency in mESCs under normal conditions. p53 is stabilized by phosphorylation and highly expressed in the nucleus of mESCs following DNA damage [12,19]. Therefore, the increased p53 expression seen following DNA damage can potentially induce mESCs to differentiate by Nanog-targeted inhibition [18]. p53 is normally expressed in the nucleus as a transcriptional factor that promotes p21 transcription, although p21 protein is not detectable in mESCs due to its rapid degradation by proteasomes [12]. It has been reported that p21 inhibits CDK2 activation and prevents G1/S cell cycle progression [20]. Moreover, p53 can also inhibit CDC25A transcription through the activation of ATF3 that is independent of p21 [21,22]. CDC25A is involved in the activation of CDK2 and facilitates G1/S transition [23]. These studies indicate that p53 can repress G1 cell cycle progression. It is now well established that p53 triggers an apoptotic response in somatic cells, but it is still uncertain whether p53 could exert a similar response in mESCs. Some studies have suggested that p53 expression is crucial for apoptotic response in mESCs [24]. In contrast, other studies have reported that p53 has little impact on apoptosis, as mESCs can undergo p53-independent apoptosis following DNA damage [19,25,26].

In this study, we investigated the role of *Babam2* in cell cycle regulation and pluripotency in mESCs after DNA damage. We also examined how these two fundamental processes were affected in *Babam2^−/−^* knockout mESCs following induced DNA damage.

## 2. Experimental Section

### 2.1. Generation of BABAM2 Knockout mESCs

Exon 5 was deleted from the *Babam2* gene to generate *Babam2^−/−^* transgenic mice as previously described [27]. *Babam2^−/−^* and wild-type (WT) mESCs were generated from the inner cell mass cells of blastocysts, harvested from the mating of heterozygous *Babam2^+/−^* mice. The preparation and manipulation of the blastocysts were performed according to modified protocols described by Nagy in 2003. Briefly, embryonic (E) 3.5 day-old blastocysts were isolated from mouse oviducts. Each blastocyst was transferred into individual wells of a 4-well culture plate containing a mouse embryonic fibroblast feeder layer that was mitotically inactivated by gamma irradiation treatment. The blastocysts were maintained in ESC culture medium (consisting of DMEM/F12 with GlutaMAXTM supplement (Gibco, Gaithersburg, MD, USA; 10565-018), 15% HyCloneTM Characterized FBS (GE Healthcare Life Sciences, Chicago, IL, USA; SH30071.03), 1× non-essential amino acids (Gibco, Gaithersburg, MD, USA; 11140-050), 1 mM sodium pyruvate (Gibco, Gaithersburg, MD, USA; 11360-070), P/S (Invitrogen, Carlsbad, CA, USA), 1× β-mercaptoethanol (Sigma-Aldrich, St. Louis, MO, USA), 1000 U/mL mouse Lif (Millipore, Burlington, MA, USA; ESG 1107), 1 μM PD0325901 (Sigma-Aldrich, St. Louis, MO, USA; PZ0162), and 3 μM CHIR99021 (Sigma-Aldrich, St. Louis, MO, USA; SML1046)) on a feeder cell layer at 37 °C in 5% CO_2_. This enables the attachment and outgrowth of the inner cell mass cells. After 5 days of culture, the inner cell mass clump from individual blastocysts was dissociated by trypsinization, replated onto a new feeder cell layer and regarded as cell passage 1. The cells were sub-cultured on a feeder layer for one more passage. Starting from passage 3, the mESCs were cultured in ESC culture medium on a gelatin-coated culture plate under feeder-free cell culture conditions. ESC culture medium was changed daily and the mESCs were sub-cultured every two days. The stably established mESC lines were then validated by genotyping.

### 2.2. Cell Proliferation Assay

*Babam2^−/−^* and WT mESCs were seeded into a 96-well culture plate at a density of 1.25 × 10^4^ cells per cm^2^ and cultured for 1 to 4 days. On the day of analysis, a 10 μL amount of cck-8 solution (Dojindo, Rockville, MD, USA) was added into each culture well along with 100 μL ESC medium and incubated for 1 h at 37 °C. Four wells each of *Babam2^−/−^* and WT mESC cultures were analyzed every day. The reactions were measured using a microplate reader (Bio-Rad Benchmark Plus, Hercules, CA, USA) with absorbance set at 450 nm.

### 2.3. Induction of DNA Damage

mESCs were seeded at 2 × 10^4^ cells per cm^2^ onto culture dishes and cultured for 2 days before treating with DNA damage-inducing agents. To induce DNA damage, the cells were exposed to gamma irradiation by placing the mESCs inside a gamma irradiator (Nordion Gammacell 3000, Ottawa, ON, Canada). The designated time of treatment was 150 s exposure, which is equivalent to 8 Gy of gamma irradiation. Doxorubicin (a chemotherapy drug) was also used to induce DNA damage. The mESCs were incubated in culture medium containing 250 nM doxorubicin for 6 h.

### 2.4. Western Blot Analysis

Western blot was performed as previously described [4]. Briefly, mESCs were harvested by scraping off the cells from the culture dishes in ice-cold cell lysis buffer (Beyotime Biotechnology, Haimen, Jiangsu, China; P0013) containing 1 mM of PMSF, 1 mM DTT, and 1× protease inhibitor (Pierce, Rockford, IL, USA). The supernatant of the cell lysate was isolated by centrifugation at 13,000 rpm for 10 min at 4 °C. The protein concentration of the supernatant was determined using a BCA Protein Assay Kit (Pierce, Rockford, IL, USA). The same quantity of protein (20 μg) was resolved in SDS-PAGE gels and transferred onto nitrocellulose membranes. The membranes were incubated with specific primary antibodies (anti-BABAM2 (Cell Signaling, Danvers, MA, USA; #12457, 1:1000), anti-β tubulin (Bio-Rad, Hercules, CA, USA; #12004166, 1:10,000), anti-OCT4 (Abcam, Cambridge, UK; ab18976, 1:1000), anti-SOX2 (Abcam, Cambridge, UK; ab92494, 1:1500), anti-NANOG (Abcam, Cambridge, UK; ab80892, 1:1000), anti-CDK2 (Cell Signaling, Danvers, MA, USA; #2546, 1:1000), anti-CDC25A (Santa Cruz Biotechnology, Dallas, TX, USA; sc-56264, 1:200), anti-p53 (Santa Cruz Biotechnology, Dallas, TX, USA; sc-71819, 1:200), anti-phospho-p53 (Ser15) (Cell Signaling, Danvers, MA, USA; #9284, 1:1000)) overnight at 4 °C, after blocking for 1 h at room temperature with 5% *w/v* non-fat dry milk in TBST (0.1% Tween-20 in TBS). The appropriate IRDye 800CW or 680RD secondary antibodies (LI-COR Biosciences, Lincoln, NE, USA, 1:10,000) were used to detect the primary antibodies and analyzed using a ChemiDoc MP Imaging System (version 5.2.1, Bio-Rad, Hercules, CA, USA).

### 2.5. Cell Cycle Profiling

For cell cycle analysis, mESC cultures were trypsinized and resuspended to obtain a single cell suspension before fixing in ice-cold 70% ethanol at 4 °C for 1 h. The cells were rehydrated in PBS before staining with 50 μg/mL propidium iodide (PI), 40 μg/mL RNase, and 0.1% Triton X-100 in PBS for 1 h in the dark. The PI-stained cells were then analyzed by flow cytometry, BD LSRFortessa Cell Analyzer (BD, Franklin Lakes, NJ, USA). The cell cycle profile of the mESCs was determined using ModFIT LT^TM^ software (version 3.0, Verity Software House, Inc., Topsham, ME, USA).

### 2.6. Apoptosis Assays

The extent of apoptosis in mESCs was determined using Annexin V-FITC and PI staining (FITC Annexin V/Dead Cell Apoptosis Kit, Invitrogen) and analyzed by flow cytometry. Gamma irradiation-treated and untreated mESCs were trypsinized and washed with PBS. Approximately 1 × 10^5^ cells in each group were incubated in 100 μL of 1× annexin-binding buffer containing 5 μL of FITC annexin V and 1 μL of 100 μg/mL PI staining solution, at room temperature for 15 min. All cells were analyzed by flow cytometry within an hour after staining. Cells that were stained Annexin V^+^ and PI^−^ represent cells at the early stage of apoptosis.

### 2.7. Immunofluorescence Staining

mESCs were seeded onto gelatin-coated 13 mm coverslips. The cells were washed with PBS and fixed in 4% paraformaldehyde for 15 min at room temperature. The fixed cells were then permeabilized using 0.1% Triton X-100 in PBS for 30 min followed by blocking with 10% horse serum in PBST (0.1% Tween-20 in PBS) for 1 h. The cells were then incubated with primary antibodies (anti-BABAM2 (Cell Signaling, Danvers, MA, USA; #12457, 1:200), anti-CDK2 (Cell Signaling, Danvers, MA, USA; #2546, 1:100), anti-phospho-p53 (Ser15) (Cell Signaling, Danvers, MA, USA; #9286, 1:400), anti-OCT4 (Abcam, Cambridge, UK; ab18976, 1:100), anti-SOX2 (Abcam, Cambridge, UK; ab92494, 1:100), anti-NANOG (Abcam, Cambridge, UK; ab80892, 1:100) made up in blocking solution at 4 °C overnight. After washing three times in PBST, specific Alexa Fluor 488 or 647-conjugated secondary antibodies (Invitrogen, Carlsbad, CA, USA; 1:5000) were used to detect the primary antibodies by incubating for 1 h at room temperature in the dark. The nuclei of the cells were then counterstained in 5 μg/mL DAPI. The fluorescence-stained cells were imaged and photographed using an FV1200 Laser Scanning Confocal Microscope (Olympus, Tokyo, Japan) and analyzed with a FluoView ver.4.2a Viewer (Olympus, Tokyo, Japan).

### 2.8. Reverse-Transcript Quantitative PCR

Total RNAs were extracted from cells using an RNeasy^®^ Plus Mini Kit (Qiagen, Hilden, Germany), according to the manufacturer’s instructions. The concentration of the RNA extract was determined using NanoDrop™ 2000/2000c Spectrophotometers (ThermoFisher Scientific, Waltham, MA, USA). The RNA was used to generate complementary DNA (cDNA) using Oligo (dT)_18_ primers with a RevertAid First Strand cDNA Synthesis Kit (ThermoFisher Scientific, Waltham, MA, USA). Real-time quantitative PCR was performed in a QuantStudio 7 (QS7) Flex Real-Time PCR System (Applied Biosystems, Foster City, CA, USA). A SYBR green reaction kit (SYBR^®^ Premix Ex Taq™, TaKaRa, Kyoto, Japan) was used for the cDNA and primer reaction that target specific genes as indicated in Table S1, Supplementary Materials. The reaction was carried out following a shuttle PCR protocol (initial denaturation: 95 °C for 30 s; 40 cycles of PCR: 95 °C for 5 s, 60 °C for 34 s; and dissociation stage: 95 °C for 15 s, 60 °C for 1 min followed by 95 °C for 15 s). A single-sharp melt curve of the PCR product was confirmed. Each set of the qPCR reaction was performed in triplicate. The relative expression of each gene was normalized against *Gapdh*, and the fold change in gene expression after treatment was normalized against the untreated control.

### 2.9. Alkaline Phosphatase Staining

Alkaline phosphatase activity in mESCs was demonstrated using an alkaline phosphatase staining kit and according to manufacturer’s instructions (Stemgent, Cambridge, MA, USA). Briefly, mESCs were fixed in 4% paraformaldehyde for 2 min and then washed with PBST. Alkaline phosphatase staining solution was prepared by mixing solutions A, B, and C from the kit at 1:1:1 ratio. The fixed cells were stained for 15 min in the dark at room temperature. The alkaline phosphatase-stained cells were photographed using an IX83 Inverted Microscope (Olympus, Tokyo, Japan).

### 2.10. Co-Immunoprecipitation

293FT cells were transfected with FLAG-BABAM2 plasmids using lipofectamine 2000. Two days after transfection, the cells were treated with 8 Gy of gamma irradiation and maintained in culture for 2 h. The cells were then lysed in lysis buffer (Beyotime Biotechnology, Haimen, Jiangsu, China) supplemented with 1 mM PMSF, 1 mM DTT, and 1× protease inhibitor tablets (Pierce, Rockford, IL, USA). Immunoprecipitation was performed by incubating precleaned cell lysate with anti-BABAM2 antibody (Cell Signaling, Danvers, MA, USA; #12457, 1:50) or anti-p53 antibody (Santa Cruz Biotechnology, Dallas, TX, USA; sc-71819, 1:50) for 4 h at 4 °C. The lysate was then added to recombinant protein G sepharose beads (ThermoFisher Scientific, Waltham, MA, USA). Immunoprecipitated proteins that have bound to the beads were collected by centrifugation at 12,000× *g* for 20 s and washed in lysis buffer 3 times. The immunoprecipitated proteins were separated from the beads by dissolving in SDS protein loading buffer and detected by Western blot analysis.

### 2.11. Ubiquitination Assay

293FT cells were transfected with HA-Ubiquitin plasmids together with or without FLAG-BABAM2 plasmids using lipofectamine 2000. Two days after transfection, the cells were exposed to 8 Gy gamma irradiation and then treated with 20 μM of MG132 for 6 h. The cells were collected in lysis buffer (Beyotime Biotechnology, Haimen, Jiangsu, China) supplemented with 1 mM PMSF, 1 mM DTT, and 1× protease inhibitor tablets (Pierce, Rockford, IL, USA). p53 was immunoprecipitated by incubating precleaned cell lysate containing anti-p53 antibody (Santa Cruz Biotechnology, Dallas, TX, USA; sc-71819, 1:50) for 4 h at 4 °C, before adding recombinant protein G sepharose beads (ThermoFisher Scientific, Waltham, MA, USA). Beads with attached immunoprecipitated proteins were collected by centrifugation at 12,000× *g* for 20 s and washed with lysis buffer 3 times. The immunoprecipitated proteins were separated from the beads by dissolving in SDS protein loading buffer, and ubiquitination of p53 was detected by Western blot analysis using HRP-tagged anti-HA antibody (Sigma-Aldrich, St. Louis, MO, USA; H6533,).

### 2.12. Statistical Analysis

Data are presented as mean ± standard deviation (SD). Statistical analysis between 2 groups was compared using Student’s unpaired two-tailed *t*-test. *p*-value of less than 0.05 was regarded as statistically significant. The *p*-value was presented in the graphs where * represents *p* < 0.05, ** *p* < 0.005, and *** *p* < 0.001.

## 3. Results

### 3.1. Absence of Babam2 Expressions Does Not Affect mESCs’ Basal Pluripotency and Proliferation

**Figure 1:**
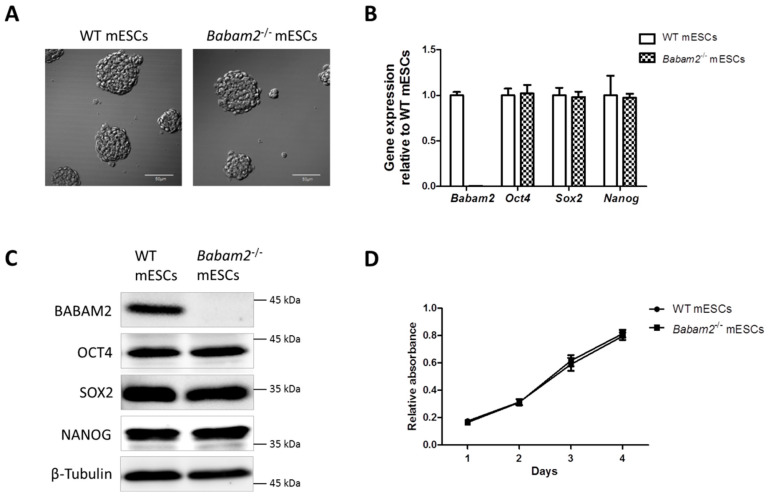
Characterization of wild-type WT and Babam2^−/−^ mouse embryonic stem cells (mESCs). (**A**) Morphology of WT and Babam2^−/−^ mESCs maintained on gelatin-coated cell culture plates under feeder-free condition. Scale bar = 50 µm. (**B**) Relative Babam2 mRNA expression and three pluripotency-associated markers in WT and Babam2^−/−^ mESCs, normalized to *Gapdh*. (**C**) Western blot analysis for expression of BABAM2 and pluripotency-associated markers in WT and Babam2^−/−^ mESCs. (**D**) Proliferation curves for WT and Babam2^−/−^ mESCs were measured as relative to cck-8 absorbance. The data show the mean of three independent experiments.

We generated embryonic stem cells (ESCs) from the blastocysts of *Babam2^−/−^* and WT mice. The mESCs were maintained on feeder-layer free conditions and they both formed round cell colonies with a clear boundary and appeared morphologically similar (Figure 1A). The absence of *Babam2* expression in *Babam2^−/−^* mESCs was confirmed by RT-qPCR and Western blot analyses (Figure 1B,C). WT and *Babam2^−/−^* mESCs expressed similar basal levels of pluripotency-associated genes, including *Oct4*, *Sox2*, and *Nanog*, at the transcript and protein levels (Figure 1B,C). There were also no significant differences in cell proliferation rate between the WT and *Babam2^−/−^* mESCs (Figure 1D).

### 3.2. Gamma Irradiation Treatment Induces Cyto-Nuclear Translocation of BABAM2 in WT mESCs

**Figure 2:**
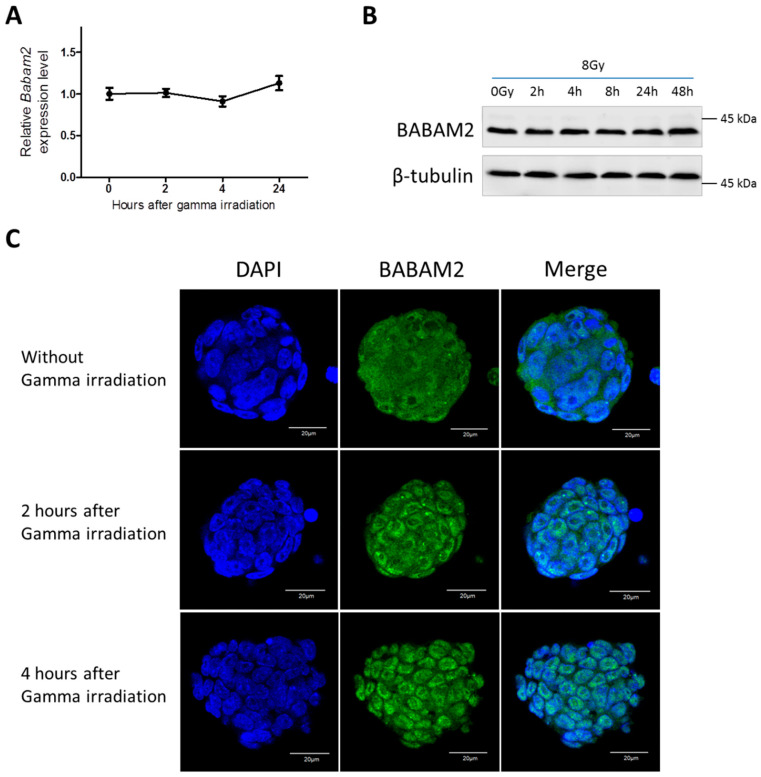
*Babam2* expression in response to gamma irradiation. (**A**) Relative *Babam2* transcription levels at various time points after gamma irradiation treatment relative to untreated control. (**B**) Western blot analysis of BABAM2 protein expression at various time points after gamma irradiation treatment. (**C**) Confocal microscopic imaging of mESCs stained with anti-BABAM2 antibodies (green) demonstrating the subcellular localization of BABAM2 in mESCs with and without gamma irradiation treatment. DAPI signal (blue) indicated for the nucleus. Scale bar = 20 µm.

It has been reported that UV radiation treatment of cancer cells inhibited *Babam2* mRNA expression [1]. Therefore, we examined *Babam2* expression in WT mESCs following gamma irradiation treatment. RT-qPCR revealed that *Babam2* expression did not significantly change over time following gamma irradiation exposure (Figure 2A). This also applied for BABAM2 protein expression (Figure 2B). We investigated how BABAM2 was spatiotemporally distributed in WT mESCs following gamma irradiation by immunofluorescence staining and confocal imaging. BABAM2 was found to be distributed in both the cytoplasmic and nuclear region before treatment. However, it began to translocate from the cytoplasm into the nucleus 2 h post-treatment, and by 4 h most of the BABAM2 proteins had translocated into the nuclei of WT mESCs (Figure 2C).

### 3.3. Prolonged G1 Phase in Babam2^−/−^ mESCs That Attenuates Gamma Irradiation-Induced Apoptotic Response

**Figure 3:**
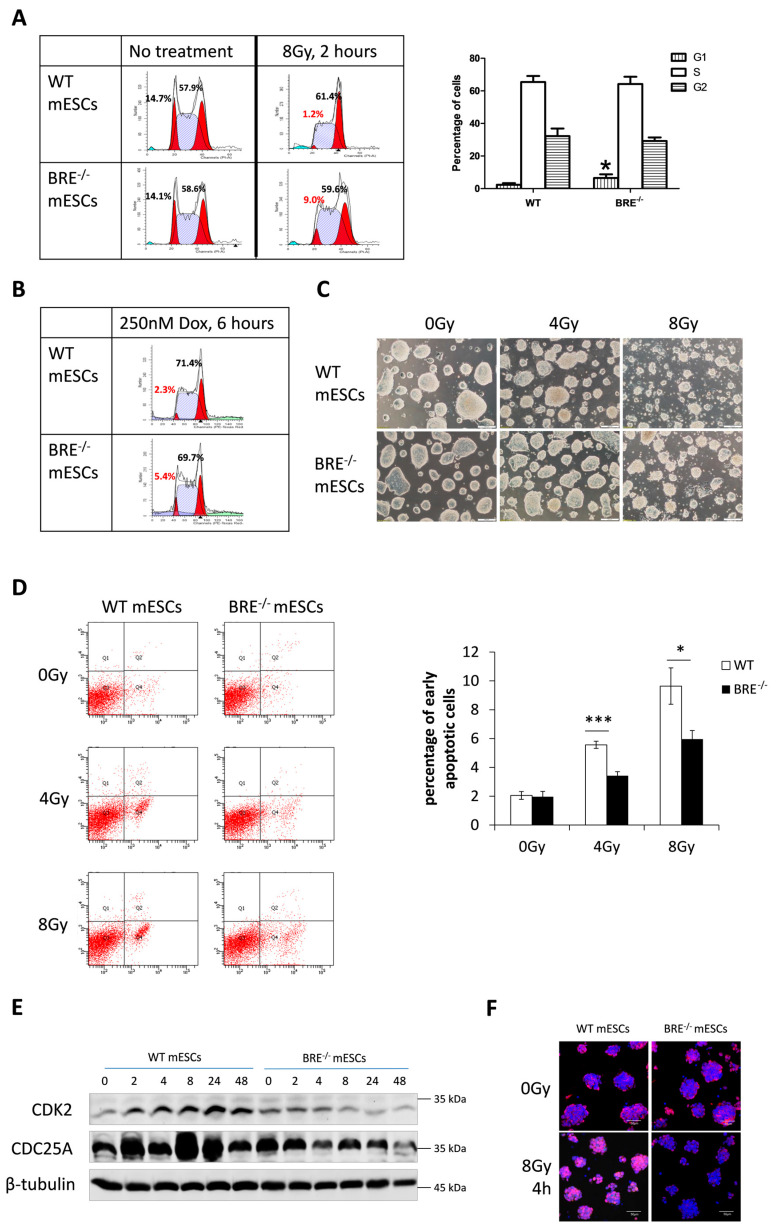
Prolonged G1 phase in Babam2^−/−^ mESCs accompanied by attenuated gamma irradiation-induced apoptotic response. (**A**) Cell cycle distribution of WT and Babam2^−/−^ mESCs analyzed by flow cytometry before and after 8 Gy of gamma irradiation treatment. The data are representative of four independent experiments. Percentages of cells at G1, S, and G2 phases 2 h after 8 Gy of treatment were measured using flow cytometry. The graph represents the mean ± SD. * indicates *p* < 0.05, two-sided unpaired *t*-test. (**B**) G1 cell accumulation in Babam2^−/−^ mESCs after 6 h of doxorubicin treatment. (**C**) Morphology of WT and Babam2^−/−^ mESCs at 24 h, after 4 and 8 Gy of gamma irradiation treatment. The data are representative of three independent experiments. Scale bar = 200 µm. (**D**) Percentage of early apoptotic cells 24 h after 4 and 8 Gy of gamma irradiation treatment as detected by Annexin V-FITC/PI staining followed by flow cytometry analysis. Data are representative of six independent experiments and mean ± SD. **, *p* < 0.005, ***, *p* < 0.001, two-sided unpaired *t*-test. (**E**) Western blot analysis of CDK2 and CDC25A expressions in response to 8 Gy of gamma irradiation treatment. (**F**) Immunofluorescence staining for CDK2 at 4 h after 8 Gy gamma irradiation treatment. DAPI (blue) stains for the nucleus. Scale bar = 50 µm.

The cell cycle profiles of gamma-irradiated *Babam2*^−/−^ and WT mESCs were determined and analyzed by flow cytometry. In samples analyzed 2 h after 8 Gy irradiation treatment, we determined that there were significantly more *Babam2*^−/−^ mESCs in G1 phase than in WT mESCs (Figure 3A). Besides gamma irradiation, doxorubicin was also used to chemically induce DNA damage. Again, a significant difference in G1 cycle response was observed between *Babam2*^−/−^ and WT mESCs after 250 nM doxorubicin treatment (Figure 3B). Therefore, the repressed G1 phase progression observed in *Babam2*^−/−^ mESCs was a general response to DNA damage and not specific to gamma irradiation treatment. Normally, the absence of G1 arrest in mESCs contributes to their hypersensitivity to DNA damage agents and protects the genomic integrity of the stem cells by eliminating any damaged cells at the G1 phase [15]. The reestablishment of the G1 checkpoint protects ESCs from DNA damage-induced apoptosis [15]. We determined that cell death was more severe in WT than in *Babam2*^−/−^ mESCs (Figure 3C). The percentage of early apoptotic cells present in WT versus *Babam2*^−/−^ mESCs was elucidated by flow cytometry, following FITC-Annexin V and PI staining. Annexin V-positive cells (while negative for PI) at Q4 of the scatter plot represents the early stage apoptotic cells. It was not surprising that *Babam2^−/−^* mESCs, with their dysregulated G1 cell cycle checkpoint, displayed significantly fewer apoptotic cells after gamma irradiation treatment (Figure 3D).

### 3.4. Decreased CDK2 and CDC25A Expressions in Babam2^−/−^ mESCs Inhibits G1 Phase Progression

High CDK2 expression enables mESCs to escape from the G1 checkpoint in the presence of induced DNA damage [14,15,28]. Our Western blot analysis revealed that CDK2 expression was significantly increased in WT mESCs, 2–48 h post-gamma irradiation treatment. In contrast, CDK2 expression was downregulated in *Babam2^−/−^* mESCs post-gamma irradiation treatment (Figure 3E). Moreover, we examined CDK2 expression and localization by immunofluorescence staining. CDK2 was normally found localized in the cytoplasm of both WT and *Babam2^−/−^* mESCs. However, it was highly expressed in the nucleus of WT mESCs, 4 h post-irradiation treatment but not in the *Babam2^−/−^* mESCs (Figure 3F). This implies that BABAM2 was essential for upregulating CDK2 to promote G1 phase progression in mESCs, in response to DNA damage.

CDC25A interacts with CDK2 upstream [15,29], so CDC25A expression was investigated by Western blot. We determined that CDC25A expression was significantly increased in WT mESCs after gamma irradiation but not in *Babam2^−/−^* mESCs (Figure 3E). This expression pattern correlated with the presence of CDK2. In addition, it indicates that BABAM2 might be involved in stabilizing and preventing CDC25A degradation upon DNA damage. Therefore, BABAM2 promotes G1 phase cell cycle progression by upregulating CDK2 through stabilizing CDC25A after gamma irradiation treatment.

### 3.5. High and Prolonged Expression of p53 in Gamma-Irradiated Babam2^−/−^ mESCs Inhibits NANOG Expression

**Figure 4:**
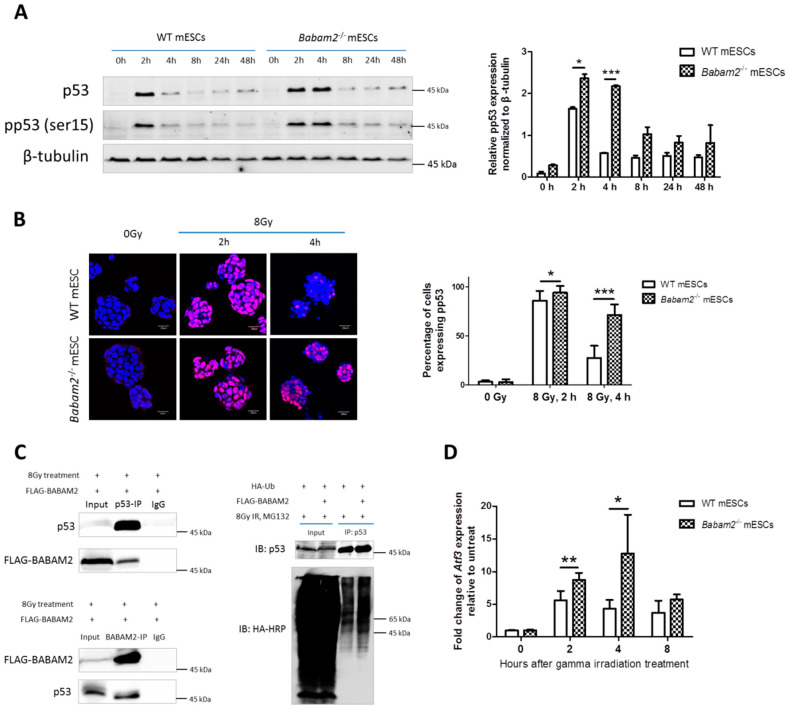
p53 are expressed at a higher level and for a longer period in Babam2^−/−^ than WT mESCs after 8 Gy gamma irradiation treatment. (**A**) Western blot showing p53 and pp53 (ser15) expressions in WT and Babam2^−/−^ mESCs at chosen time points after 8 Gy treatment. High expressions of p53 and pp53 were observed 4 h after gamma irradiation in Babam2^−/−^ mESCs but not in WT mESCs. Relative expression of pp53 normalized against β-tubulin is presented in the bar chart. Data represent the mean ± SD of three independent experiments. * indicates *p* < 0.05, two-sided unpaired *t*-test. *** indicates *p* < 0.001, two-side unpaired *t*-test. (**B**) Immunofluorescence staining for pp53 in Babam2^−/−^ and WT mESCs with or without gamma irradiation treatment. The nuclei were counterstained with DAPI. Scale bar = 20 µm. At least 300 cells from 10 colonies of mESCs were analyzed. The graph represents the mean ± SD. * indicates *p* < 0.05, two-sided unpaired *t*-test. ** indicates *p* < 0.001, two-sided unpaired *t*-test. (**C**) Interaction between p53 and overexpressed FLAG-BABAM2 was confirmed by co-immunoprecipitation of BABAM2 with the p53-specific antibody. p53 was also co-immunoprecipitated with overexpressed FLAG-BABAM2 using anti-BABAM2 antibodies in the 293FT cell line. p53 ubiquitination in 293FT cells with and without BABAM2 overexpression was analyzed by Western blot analysis and detected with HRP-tag anti-HA antibodies. (**D**) RT-qPCR analysis revealed the fold change in the p53-targeted gene, Atf3, expression relative to untreated Babam2^−/−^ and WT mESCs after 8 Gy treatment. Data represent the mean ± SD of three independent experiments. *, *p* < 0.05, **, *p* < 0.005, two-sided unpaired *t*-test.

We investigated whether BABAM2 absence affected p53 response to gamma irradiation treatment. Western blot analysis revealed p53 and pp53 expressions were rapidly induced 2 h after irradiation exposure and that they were significantly stronger in Babam2^−/−^ than WT mESCs (Figure 4A). Nevertheless, p53 and pp53 expressions were rapidly degraded after 4 h in WT mESCs but still remained highly expressed in *Babam2*^−/−^ mESCs. Immunofluorescence staining revealed pp53 was localized in the nucleus and concurred with the Western blot results (Figure 4B). The percentage of cells expressing pp53, 2 h after 8 Gy treatment, were significantly higher in *Babam2*^−/−^ mESCs (* and *** denote *p* < 0.05 and < 0.001, respectively, two-sided unpaired *t*-test). We investigated the reasons for the relatively prolonged and higher level of p53 expression in *Babam2*^−/−^ mESCs. We reasoned that BABAM2 and p53 might interact with each other. To determine whether this was the case, we decided to gamma irradiate 293FT cells to induce p53 expression. The lysates from these cells were then co-immunoprecipitated with BABAM2- or p53-specific antibodies. Western blots revealed that p53 could interact with BABAM2 as confirmed by co-immunoprecipitation of FLAG-BABAM2 with p53 using p53-specific antibody (Figure 4C, left upper panel). Moreover, co-immunoprecipitation of p53 with BABAM2 using BABAM2-specific antibodies (Figure 4C, left lower panel). We also demonstrated that overexpression of BABAM2 could increase p53 ubiquitination (Figure 4C, right panel), indicating that BABAM2 promotes p53 ubiquitination. Moreover, prolonged p53 expression in *Babam2*^−/−^ mESCs significantly induced *Atf3* expression at 2 and 4 h post-gamma irradiation (Figure 4D).

**Figure 5:**
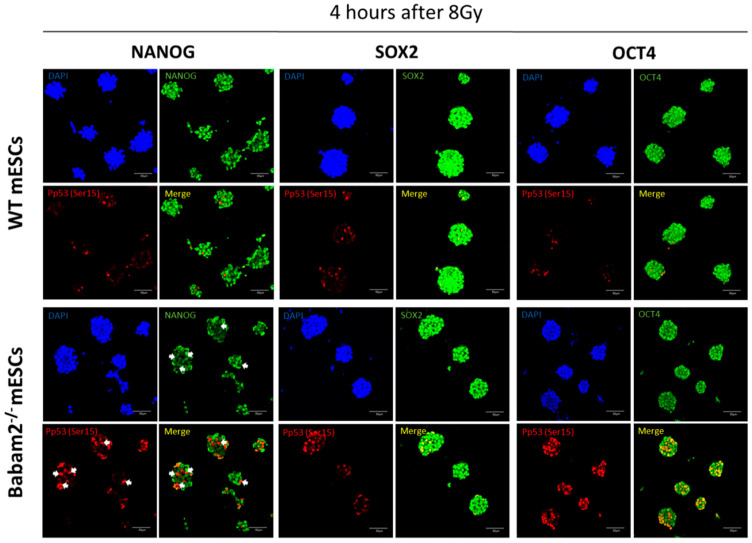
pp53 inhibits NANOG expression in mESCs. Co-immunofluorescence staining of WT and Babam2^−/−^ mESCs at 4 h after 8 Gy of gamma irradiation for pp53 (ser15) with NANOG, SOX2, and OCT4, respectively. Cells expressing pp53 do not express NANOG (as indicated by the white arrows) but still express SOX2 and OCT4. Scale bar = 50 µm.

We used co-immunofluorescence staining to investigate p53, OCT4, SOX2, and NANOG expressions, 4 h post-irradiation treatment (Figure 5). Immunofluorescence imaging revealed that pp53 expressions were almost abolished in WT mESCs but still remained strongly expressed in nuclei of *Babam2*^−/−^ mESCs. We then examined the consequence of high pp53 expressions in *Babam2*^−/−^ mESCs and found they inhibited NANOG expression but not OCT4 and SOX2. In contrast, NANOG, OCT4, and SOX2 expressions remained high in WT mESCs, whereas pp53 expression was low.

### 3.6. BABAM2 Is Essential for Maintaining Pluripotency in mESCs after Gamma Irradiation Treatment

**Figure 6:**
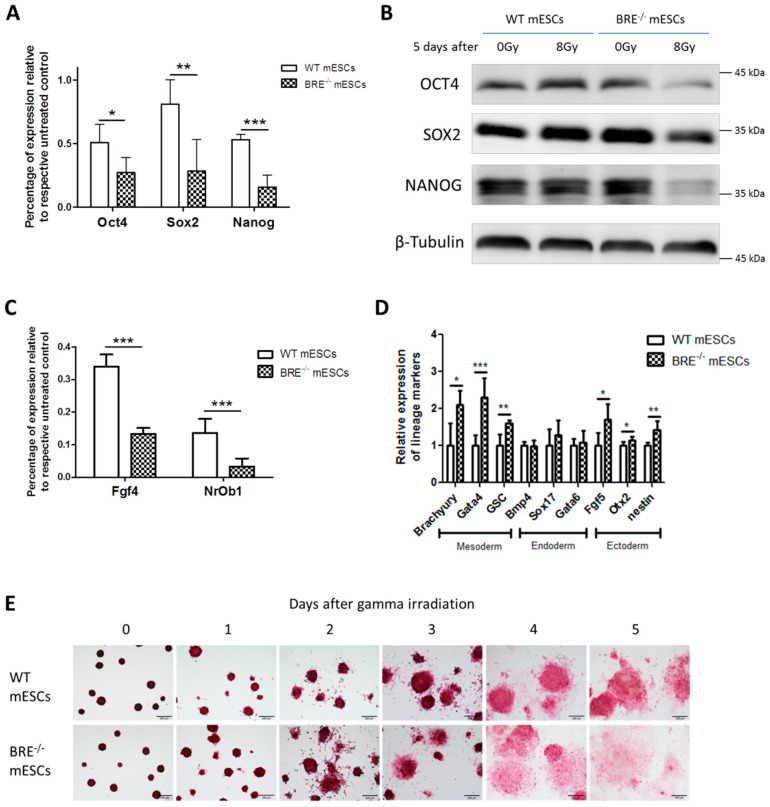
Expressions of pluripotency-associated genes were sufficiently downregulated in Babam2^−/−^ mESCs after 8 Gy of gamma irradiation treatment. (**A**) Relative protein expression of pluripotency-associated genes in 5 days post-irradiation (8 Gy) treated and untreated Babam2^−/−^ and WT mESCs. Data represent the mean ± SD of three independent experiments. *, *p* < 0.05, **, *p* < 0.005, ***, *p* < 0.001, two-sided unpaired *t*-test. (**B**) Western blot analysis of pluripotent markers, OCT4, SOX2, and NANOG, in Babam2^−/−^ and WT mESCs, 5 days after 8 Gy treatments. (**C**) Relative mRNA expression of naïve stem cell markers in 5 days post-irradiation (8 Gy) treated and untreated Babam2^−/−^ and WT mESCs. Data represent the mean ± SD of three independent experiments. ***, *p* < 0.001, two-sided unpaired *t*-test. (**D**) Relative mRNA expression level of differentiation gene markers, normalized against Gapdh in Babam2^−/−^ and WT mESCs 5 days post-irradiation. Data represent the mean ± SD of three independent experiments. *, *p* < 0.05, **, *p* < 0.005, ***, *p* < 0.001, two-sided unpaired *t*-test. (**E**) Representative images of alkaline phosphatase staining of Babam2^−/−^ and WT mESCs at different day intervals following 8 Gy of gamma irradiation treatment. Scale bar = 200 µm.

We showed that gamma irradiation induced a significantly stronger and more prolonged pp53 response in *Babam2^−/−^* than WT mESCs. In addition, it inhibited NANOG expression. Hence, we wanted to further investigate the long-term effects of gamma irradiation on *Babam2^−/−^* pluripotency. This was realized by exposing WT and *Babam2^−/−^* mESCs to 8 Gy of irradiation and then maintaining the cells for 1 to 5 days in culture. RT-qPCR revealed that *Babam2^−/−^* mESCs significantly reduced their expression of pluripotency-associated genes, *Oct4*, *Sox2*, and *Nanog*, as compared with WT mESCs (Figure 6A). Western blot analysis also validated the RT-qPCR results by showing OCT4, SOX2, and NANOG expressions were significantly reduced in *Babam2^−/−^* mESCs, 5 days post-irradiation treatment (Figure 6B). Moreover, there was reduced expression of naïve stem cell markers, *fgf4* and *NrOb1* (Figure 6C), and increased expression of mesodermal and ectodermal differentiation markers including *Brachyury*, *Gata4*, *GSC*, *Fgf5*, *Otx2*, and *Nestin* observed in *Babam2^−/−^* mESCs as compared with WT mESCs (Figure 6D).

Alkaline phosphatase activity is associated with stemness and pluripotency in ESCs [30]. Hence, we examined the activities of this enzyme in *Babam2^−/−^* and WT mESCs, 1–5 days post-irradiation treatment. Alkaline phosphatase staining revealed that *Babam2^−/−^* mESCs lost their alkaline phosphatase activity significantly faster than WT mESCs, 3–5 days post-irradiation treatment (Figure 6E). The results indicate that BABAM2 was required to maintain pluripotency in mESCs in response to gamma irradiation exposure.

## 4. Discussion

Previously, we have shown that loss of Babam2 in fibroblasts leads to proliferation defects and premature cellular senescence [4]. Although pluripotent mESCs are known to have unlimited self-renewal capability without entering senescence, we demonstrated that loss of Babam2 in mESCs inhibited cell cycle progression after DNA damage, accompanied by CDC25A degradation. We also found that CDK2, a downstream target of CDC25A, expression was simultaneously inhibited in *Babam2^−/−^* mESCs. These mESCs have a significantly reduced proliferation rate and expression of pluripotent gene markers but an increased one in trophectodermal differentiation-associated markers. Moreover, it has been established that CDK2 readily phosphorylates and stabilizes pluripotent-associated NANOG, OCT4, and SOX2 proteins [16,17]. In this context, the prolonged G1 phase and reduced CDK2 expression in *Babam2^−/−^* mESCs following DNA damage together contribute to a loss of pluripotency in *Babam2^−/−^* mESCs.

We found that gamma irradiation treatment of WT mESCs rapidly induces p53 expression and phosphorylation, but p53 is then rapidly degraded, in the space of 2 h. In contrast, irradiation-induced p53 expression was significantly higher and more prolonged in *Babam2^−/−^* mESCs. It is known that p53 can block the G1/S phase cell cycle progression by inhibiting CDC25A through the activation of *Atf3* [21,22]. Consequently, we analyzed *Atf3* expression following gamma irradiation and found that *Atf3* expression was significantly elevated in *Babam2^−/−^* mESCs as compared with their normal counterpart. This finding indicates that the irradiation-treated *Babam2*^−/−^ mESCs, with their higher p53 expression level, may also inhibit CDC25A through the activation of *Atf3*. Simultaneously, the prolonged and higher level of p53 expression may also contribute to CDK2 inhibition observed in *Babam2^−/−^* mESCs after DNA damage. p21 is another downstream effector of p53 and a well-known cyclin-dependent kinase (Cdk) inhibitor [20]. However, p21 protein was barely detected in mESCs even after irradiation [12] and in our mESCs. Therefore, p53 should regulate cell cycle progression in mESCs mainly through the Atf3-Cdc25a pathway.

p53 has been reported to suppress *Nanog* transcription in mESCs [18]. We further confirmed that p53 inhibited NANOG protein expression, but it did not directly inhibit OCT4 and SOX2 in mESCs. *Oct4*, *Sox2*, and *Nanog* normally modulate each other’s expressions as a core circuit regulating pluripotency in ESCs [31,32]. Overexpression of *Nanog* alone in mESCs can maintain stem cell identity, cell colony-forming ability, and inhibition of differentiation—even after the removal of LIF [33]. Inversely, the loss of NANOG expression in mESCs would lead to a very low level of OCT4 expression, significantly reducing cell proliferation, and the cells would develop a flattened-differentiated morphology [34]. Furthermore, a comprehensive study on gene binding and regulation by NANOG revealed that NANOG controls *Oct4* and *Sox2* expressions [35]. All of these studies indicate that *Nanog* plays a vital role in regulating the core circuits that maintain pluripotency. Abbreviated NANOG expression could account for the reduced expression of pluripotency markers OCT4 and SOX2 following targeted inhibition of NANOG by p53 in mESCs, in response to DNA damage. For this reason, *Babam2^−/−^* mESCs have a lower pluripotency status and higher expression of differentiation markers as compared with WT mESCs. Remarkably, NANOG in return promotes G1/S cell cycle progression by promoting CDC25A expression [36].

**Figure 7:**
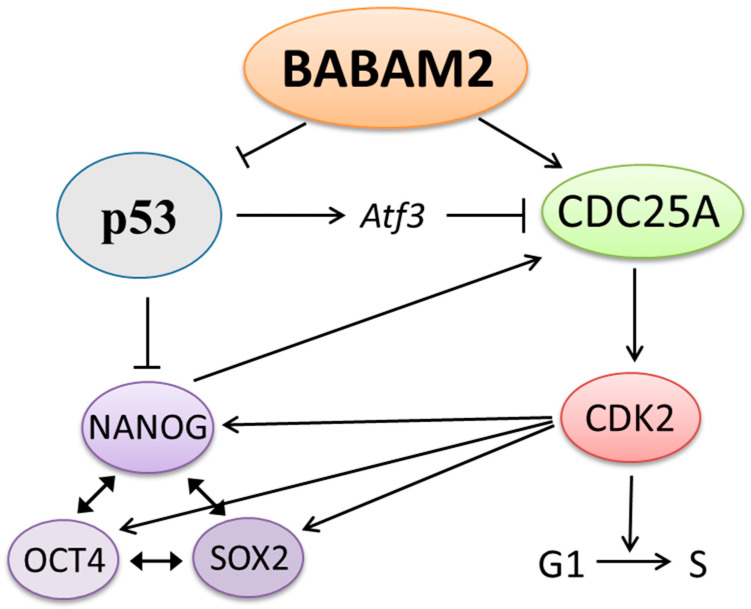
Schematic diagram showing BABAM2’s involvement in the regulation of cell cycle progression and pluripotency in mESCs following DNA damage. BABAM2 stabilizes CDC25A by promoting the deubiquitination of CDC25A. CDC25A then activates CDK2 and promotes G1/S cell cycle progression. In addition, CDK2 stabilizes NANOG, OCT4, and SOX2, which are key regulators of the pluripotency state. BABAM2 can inhibit p53 by promoting p53 ubiquitination. p53 can inhibit cell cycle progression by inhibiting CDC25A through the activation of Atf3. In addition, p53 can inhibit NANOG expression that plays an important role in regulating the core circuits of pluripotency. NANOG has also been reported to promote CDC25A expression.

Interestingly, BABAM2 has two ubiquitin-conjugating enzyme family-like regions containing no active sites, suggesting that BABAM2 might act as a co-factor regulating the ubiquitination of specific target proteins. Indeed, BABAM2 is involved in regulating the ubiquitination of CDC25A by interacting with USP7 [5] and interacting with p53 to promote p53 ubiquitination [6]. Both p53 and CDC25A act together to regulate G1/S cell cycle progression and pluripotency in mESCs after DNA damage. In this study, we clearly demonstrated that a prolonged and elevated level of p53 expression is linked with the inhibition of CDC25A and CDK2 expressions in *Babam2^−/−^* mESCs. We also provided direct evidence indicating that BABAM2 is essential for maintaining proper cell cycle regulation and pluripotency in mESCs following DNA damage. In Figure 7, we summarize the interdependent molecular pathways that BABAM2 is involved in the regulation of cell cycle progression and maintenance of pluripotency. In conclusion, our study provides functional evidence that BABAM2 plays an important role in regulating cell cycle progression and pluripotency status following induced DNA damage.

## Supplementary Materials

The following are available online at https://www.mdpi.com/2227-9059/8/10/397/s1, Table S1: Primer sequences for RT-qPCR.

## Abbreviations

Babam2	BRISC and BRCA1-A complex member 2
BRE	Brain and reproductive organ-expressed
WT	Wild-type
mESCs	Mouse embryonic stem cells
CDC25A	Cell division cycle 25 A
CDK2	Cyclin-dependent kinase 2
UEV-like	Ubiquitin-conjugating enzyme family-like
USP7	Ubiquitin-specific-processing protease 7
Mdm2	Mouse double minute 2
OCT4	Octamer-binding transcription factor 4
SOX2	Sex-determining region Y-box 2
NANOG	Nanog homeobox
p53	Protein 53
p21	Cyclin-dependent kinase inhibitor 1A
ATF3	Activating transcription factor 3
Gata4	GATA binding protein 4
*GSC*	Goosecoid homeobox
*Fgf5*	Fibroblast growth factor 5
*Otx2*	Orthodenticle homeobox 2
HA	Hemaglutinin antigen
RT-qPCR	Reverse transcription-quantitative polymerase chain reaction
cDNA	Complementary DNA
PBS	Phosphate buffered saline
PBST	0.1% Tween-20 in PBS
PMSF	Phenylmethylsulfonyl fluoride
DTT	Dithiothreitol
SD	Standard deviation
