# Supplementary material for: Babam2 Regulates Cell Cycle Progression and Pluripotency in Mouse Embryonic Stem Cells as Revealed by Induced DNA Damage
Source: Biomedicines. 2020 Oct 10;8(10):397. doi: 10.3390/biomedicines8100397 (PMC7600899; doi:10.3390/biomedicines8100397)
Supplement: Supplementary file 1 [file biomedicines-08-00397-s001.pdf]

Supplymentary Table S1. Primer sequences for RT-qPCR.

Name of gene	Forward primer (5’ to 3’)	Reverse primer (5’ to 3’)
Gapdh	TGTGTCCGTCGTGGATCTGA	TTGCTGTTGAAGTCGCAGGAG
BRE	TGCAGAAGGCTTCACAAAAC	AAAAACAGGGGCAGGTCAAT
Oct4	ACCCTGGGCGTTCTCTTT	GTTGTCGGCTTCCTCCAC
Sox2	TAGAGCTAGACTCCGGGCGATGA	TTGCCTTAAACAAGACCACGAAA
Nanog	CAGGTGTTTGAGGGTAGCTC	CGGTTCATCATGGTACAGTC
Fgf4	GCAAGCTCTTCGGTGTGC	CGTAGGATTCGTAGGCGTTG
NrOb1	CAGGCCATCAAGAGTTTCTTTT	CCTCAATGTATTTCACGCACTG
Atf3	TGAGCCACTTTGTGCCAACA	TGTGCCCAGGGTTCTTCCT
